# The Effect of In-Ear and Behind-Ear Transcutaneous Auricular Vagus Nerve Stimulation on Autonomic Function: A Randomized, Single-Blind, Sham-Controlled Study

**DOI:** 10.3390/jcm13154385

**Published:** 2024-07-26

**Authors:** Alper Percin, Ali Veysel Ozden, Semiha Yenisehir, Berkay Eren Pehlivanoglu, Ramazan Cihad Yılmaz

**Affiliations:** 1 Department of Physiotherapy and Rehabilitation, Faculty of Health Sciences, Avrasya University, 61080 Trabzon, Turkey; 2 Department of Physiotherapy and Rehabilitation, Faculty of Health Sciences, Bahcesehir University, 34330 Istanbul, Turkey; aliveysel.ozden@bau.edu.tr (A.V.O.); berkayeren.pehlivanoglu@bau.edu.tr (B.E.P.); 3 Department of Physiotherapy and Rehabilitation, Faculty of Health Sciences, Mus Alparslan University, 49250 Mus, Turkey; s.yenisehir@alparslan.edu.tr; 4 Department of Physiotherapy and Rehabilitation, Faculty of Health Sciences, Igdır University, 76000 Igdır, Turkey; rcihad.yilmaz@igdir.edu.tr

**Keywords:** autonomic nervous systems, heart rates, diastolic pressure, systolic pressure, vagus nerve stimulations

## Abstract

**Background/Objectives:** Transcutaneous auricular vagus nerve stimulation (TaVNS) is a non-invasive method of electrical stimulation used to autonomic neuromodulation. Position and form of the electrodes are important for the effectiveness of autonomic modulation. This study was aimed to investigate the effect of TaVNS in-ear and behind-ear on autonomic variables. **Methods**: A total of 76 healthy participants (male: 40, female: 36) were randomized into four groups as in-ear TaVNS, behind-ear TaVNS, in-ear sham, and behind-ear sham. The TaVNS protocol included bilateral auricular stimulation for 20 min, 25 hertz frequency, a pulse width of 250 μs, and a suprathreshold current (0.13–50 mA). Heart rate (HR), systolic and diastolic blood pressure (SBP and DBP), and heart rate variability (HRV) were measured baseline and after stimulation. The parameters RMSSD (root mean square of consecutive differences between normal heartbeats), LF power (low-frequency), and HF power (high-frequency) were assessed in the HRV analysis. **Results**: HR decreased in the in-ear TaVNS after intervention (*p* < 0.05), but did not change in behind-ear TaVNS and sham groups compared to baseline (*p* > 0.05). SBP and DBP decreased and RMSSD increased in the in-ear and behind-ear TaVNS groups (*p* < 0.05), but did not change in sham groups compared to baseline (*p* > 0.05). There was no significant difference in LF and HF power after TaVNS compared to baseline in all groups (*p* > 0.05). SBP was lower and RMSSD was higher in-ear TaVNS than behind-ear TaVNS after intervention (*p* < 0.05). **Conclusions**: In-ear TaVNS appears to be more effective than behind-ear TaVNS in modulating SBP and RMSSD, but this needs to be studied in larger populations.

## 1. Introduction

Transcutaneous auricular vagus nerve stimulation (TaVNS) is a low-risk, low-cost treatment modality that can be used in a wide range of conditions, including neurology, psychiatry, cardiology, and rheumatology, as well as for the management of symptoms caused by autonomic nervous system (ANS) disorders [1,2,3,4,5]. TaVNS, which is characterized by electrical stimulation of the outer ear, can have antidepressant, antiepileptic, anti-inflammatory, cardiac modulatory, and analgesic effects by modulating the vagus nerve [6,7,8]. Related to the neuromodulator activity of ANS, TaVNS may be a preferred therapeutic modality for the control of chronic diseases associated with unstable ANS [9].

The vagus nerve is the longest nerve that provides parasympathetic innervation to the autonomic nervous system and maintains sensory and motor function by extending along the neck, thorax, and abdominal cavity [10]. In the external auditory sensory region, the auricular branch of the vagus nerve has been found to innervate the tragus around the auditory meatus, the concha, the inner surface of the external auditory canal, and the posterior wall of the external auditory canal [11]. The direct relationship of the vagus nerve to the nucleus tractus solitarii (NTS) in the spinal cord facilitates the transmission of signals received from the outer ear with afferent nerve endings to the cerebral cortex and the modulation of the ANS [12].

Motor fibers of the vagus nerve are responsible for innervation of the atria, sinoatrial node, atrioventricular node, ventricular myocardium, and ventricular conduction system [13]. Preganglionic fibers of the vagus nerve synapse with parasympathetic ganglia in the epicardium, atria, and ventricular septum [14]. TaVNS is thought to exert a negative chronotropic effect on cardiac pacemaker cells, causing acetylcholine release and hyperpolarization of the cell membrane potential by binding acetylcholine to muscarinic receptors [15]. The parasympathetic control mechanism of the heart via the vagus nerve results in heart rate (HR) and blood pressure oscillations in response to external stimuli [16]. Therefore, cardiovascular responses, including HR, heart rate variability (HRV), systolic blood pressure (SBP), and diastolic blood pressure (DBP), are assessed to clearly evaluate the therapeutic effects of TaVNS [17].

In this study, it was hypothesized that TaVNS would have effects similar to those of in-ear TaVNS, given the distribution of vagus nerve fibers behind the ear. The aim of this study was to compare the effects of in-ear and behind-the-ear TaVNS on HR, SBP, DBP, and HRV to determine which site of stimulation is most effective.

## 2. Materials and Methods

### 2.1. Study Design

This prospective, single-center, assessor-blind, sham-controlled, and parallel group design clinical study was conducted at the Physical Therapy Laboratory of the Faculty of Health Sciences, Iğdır University between October and December 2023, in accordance with the rules of the Declaration of Helsinki. The participants were asked to sign the informed consent form before participating in the study. The clinical trial registration number was NCT06312293.

### 2.2. Participants

The study included 76 healthy participants aged between 18–45 years who could read and write Turkish. The inclusion criteria were as follows: being 18 years or older, having no known acute or chronic disease, and no previous treatment with TaVNS. The exclusion criteria were previous vagotomy, myocardial infarction or arrest, cardiac conduction disorders, intracranial hemorrhage, history of cancer, using any medication supplement, and pregnancy during the study period. Participants were asked to abstain from alcohol, caffeine, and smoking for at least two hours prior to the study.

### 2.3. Study Protocol

An allocation sequence of random numbers between 1 and 76, by an independent researcher using computer software (Version 4.0) (www.randomizer.org, accessed on 1 October 2023), was used to randomize participants into four groups. The interventions were applied by another researcher. Only the assessor was not aware of the group to which the participant was allocated. The participants and physiotherapist were not blinded due to the nature of intervention.

The first group received in-ear TaVNS (n = 19), the second group received behind-the-ear TaVNS (n = 19), the third group received in-ear sham TaVNS (n = 19), and the fourth group received behind-the-ear sham TaVNS (n = 19).

HR, blood pressure, and HRV were measured in each group at baseline and after the intervention. HR and blood pressure measurements were repeated twice, and the mean of the results was calculated.

### 2.4. Assessment

#### 2.4.1. Heart Rate and Blood Pressure

The HR, SBP, and DBP values were measured at baseline and after TaVNS using an Omron M2 digital blood pressure monitor (© 2024 OMRON Healthcare, Inc., Vernon Hills, IL, USA). The measurements were performed from both the right and left arms at baseline and after intervention to ensure the reliability [18]. The mean of the values obtained was taken. Participants were verbally informed to avoid high-calorie foods, caffeinated beverages, or smoking within two hours prior to the measurements.

#### 2.4.2. Heart Rate Variability

The HRV of the participants was measured with a Polar H7 device (Copyright 2016 Polar Electro Oy, FI-90440 Kempele, Espoo, Finland). Polar H7 device is compliant with directives 2014/53/EU and 2011/65/EU. Polar Electro Oy is an ISO Standard No. 9001:2008 (Kempele, Finland, 2016) certified company.

Firstly, the Polar H7 device was placed at heart level with a chest strap and the data obtained from this device were synchronized and transferred to the Polar Flow application (© Polar Electro, 2024) via a Bluetooth-connected iPhone SE (third generation) in the HRV measurement process. In this step, a five-minute measurement was preferred [19]. Secondly, the data transferred to the Polar Flow application were analyzed with Kubios HRV standard software (version 3.5.0) (www.kubios.com, accessed on 5 October 2023). Related to outputs provided by the Kubios HRV Standard software, the root mean square values of the successive differences between normal heartbeats (RMSSD), low-frequency (LF) power, and high-frequency (HF) power were recorded at baseline and after TaVNS.

Participants were verbally informed to avoid high-calorie foods, caffeinated beverages, or smoking within two hours prior to HRV measurement. All measurements were performed in a quiet room with a room temperature of 20–22 °C (68–77 °F) in a seated position.

### 2.5. Intervention

**Figure 1:**
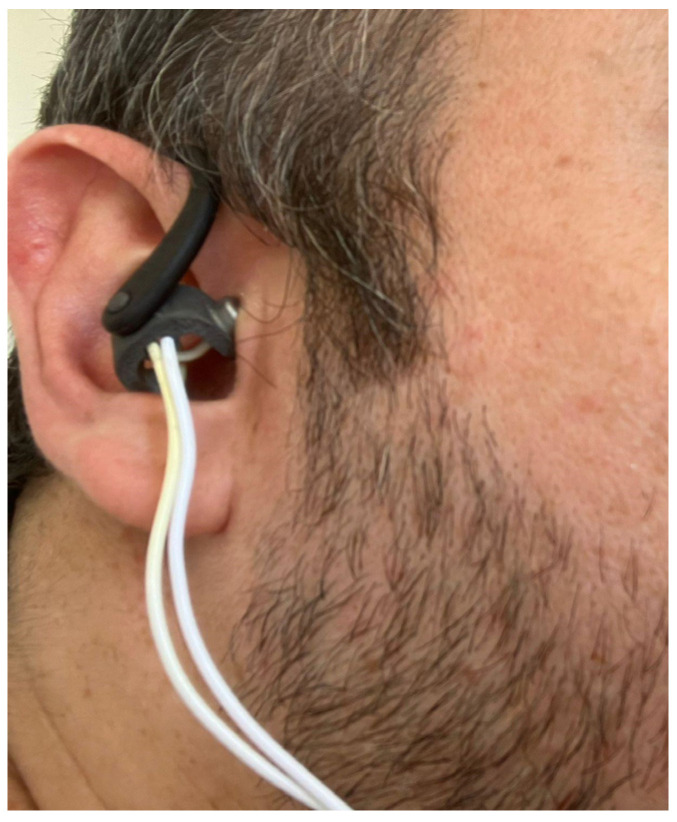
Vagustim earsets.

For in-ear TaVNS, bilateral earsets (Copyright Vagustim, 2023, Vagustim Health Technologies, San Francisco, CA, USA) with a surface area of 36 square millimeters were used to stimulate the tragus and concha (Figure 1). The Vagustim device was the preferred stimulator (Copyright Vagustim, 2023, Vagustim Health Technologies, San Francisco, CA, USA).

**Figure 2:**
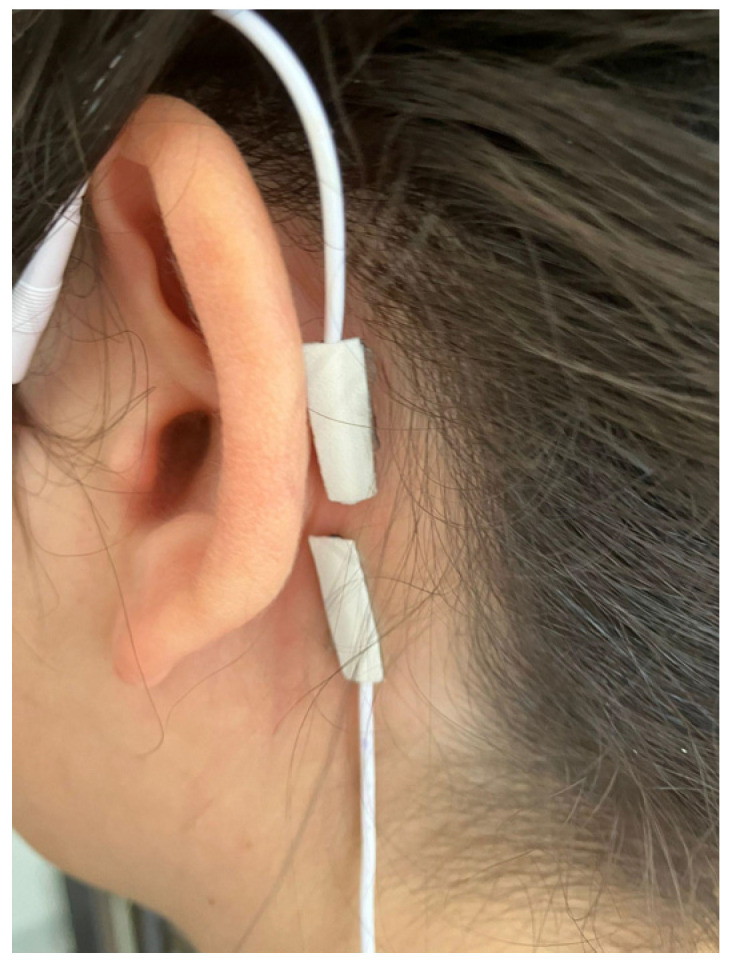
Electrode pads for behind-ear TaVNS.

For behind-ear TaVNS, adhesive electrode pads were shaped and designed to be 36 square millimeters (3 mm × 12 mm) to use a similar surface area (Figure 2). The Vagustim device was also preferred as a stimulator.

The TaVNS protocol included bilateral auricular stimulation for 20 min, a stimulation frequency of 25 hertz (Hz), a pulse width of 250 μs, a suprathreshold current (0.13–50 mA), and biphasic mode [20].

Participants were also randomized to the in-ear and behind-ear sham intervention groups using the same Vagustim device. The same specially designed electrodes were placed in-ear and behind-ear. The duration of the session was 20 min. The device opened but the amplitude was set to 0 mA so that no electrical stimulation occurred.

### 2.6. Statistical Analysis

Sample size was calculated by using G-Power (MAC version 3.1.9.6, Heinrich-Heine Universität Düsseldorf, Düsseldorf, Germany) (F test, repeated measures, between factors) based on HRV and blood pressure measurements. When F test family and ANOVA: repeated measures, within–between interaction test, effect size: 0.25, alpha level: 0.05, power (1-β): 0.95, number of groups: 4, number of measurements: 2, correction among repeated measures: 0.5 was selected, the total sample size was calculated as 76 participants (19 participants in each group).

All the statistical analyses were performed using IBM SPSS software (version 28.0.1.0, Chicago, IL, USA). The Shapiro—Wilk test was used to determine whether the variables in the study were normally distributed. Normally distributed variables are presented as the mean and standard deviation (SD). The paired samples *t*-test was used to compare dependent groups when the parametric assumption was met. The change in the dependent variables compared to the independent variables was determined using ANOVA. One-way repeated measures ANOVA and post hoc tests were used for comparisons between groups. Partial eta squared (ηp^2^) were used as an estimate of effect size, and values of 0.01, 0.06, and 0.14 were interpreted as small, medium, and large effects, respectively. Results were corrected for multiple comparisons with false discovery rate (FDR) correction. Statistical significance was considered at *p* < 0.05 and a 95% confidence interval.

## 3. Results

**Figure 3:**
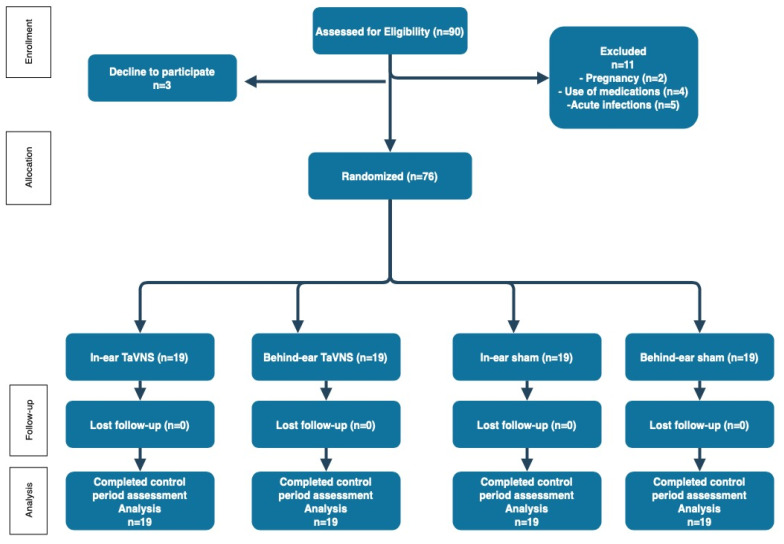
Flow diagram of study.

The number of participants randomized and analyzed for each group is shown in the flowchart (Figure 3).

### 3.1. Physical Characteristics

A total of 76 participants completed the study, and the baseline physical characteristics of the participants are shown in Table 1. At baseline, the groups were similar according to physical characteristics (*p* > 0.05).

### 3.2. Heart Rate

Changes in HR at baseline and after TaVNS are shown in Table 2. HR significantly decreased in the in-ear TaVNS group after TaVNS (*p* < 0.05) compared to baseline. There was no significant difference in HR after TaVNS in the behind-ear TaVNS, in-ear sham, and behind-ear sham groups compared to baseline (*p* > 0.05).

### 3.3. Blood Pressure

Changes in SBP and DBP at baseline and after TaVNS are shown in Table 2. After TaVNS, SBP decreased significantly in the in-ear TaVNS group (*p* < 0.05) and the behind-ear TaVNS group compared to baseline (*p* < 0.05). There was no significant difference in SBP after TaVNS between the in-ear sham and behind-ear sham groups compared to baseline (*p* > 0.05).

After TaVNS, DBP significantly decreased in the in-ear TaVNS and the behind-ear TaVNS groups compared to baseline (*p* < 0.05). There was no significant difference in DBP after TaVNS in the in-ear sham and behind-ear sham groups compared to baseline (*p* > 0.05) (Table 2).

### 3.4. Heart Rate Variability

The changes in the RMSSD, LF power, and HF power (HRV sub-parameters) at baseline and after TaVNS are shown in Table 2.

The RMSSD significantly increased in the in-ear TaVNS and behind-ear TaVNS groups after TaVNS compared to baseline (*p* < 0.05). There was no significant difference in the RMSSD after TaVNS between the in-ear sham and behind-the-ear sham groups compared to baseline (*p* > 0.05) (Table 2).

There was no significant difference in LF and HF power after TaVNS in-ear TaVNS, behind-ear TaVNS, in-ear sham, and behind-ear sham groups compared to baseline (*p* > 0.05) (Table 2).

### 3.5. Comparison of the Groups

The comparisons of the HR, SBP, DBP, RMSSD, LF power, and HF power between the groups at baseline and after TaVNS are shown in Table 3.

There was no statistical difference between groups at the baseline (*p* > 0.05) (Table 3). No significant difference was found in HR after TaVNS between all groups (*p* > 0.05). A significant difference between the groups was found for SBP with large effect size by partial eta squared measure and RMSSD with medium effect size by partial eta squared measure after TaVNS (*p* < 0.05) (Table 3).

**Figure 4:**
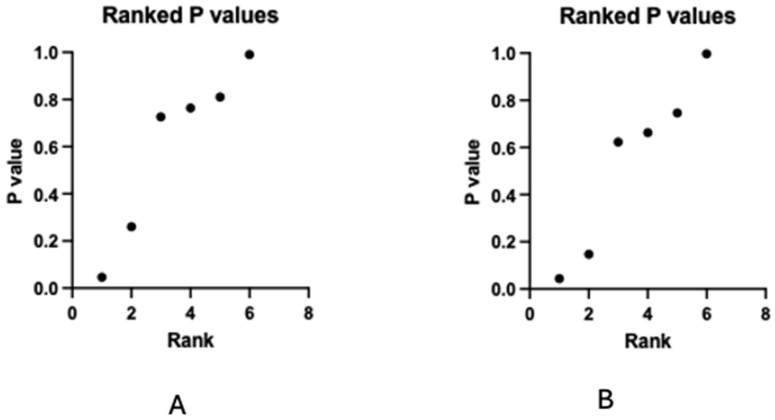
Corrected *p*-values with false discovery rate (FDR) after post hoc analysis. (**A**) RMSSD, (**B**) systolic blood pressure.

The post hoc analysis participants showed rate of change differences in terms of a decrease in SBP and an increase in RMSSD (Table 4). SBP was lower and RMSSD was higher in-ear TaVNS than behind-ear TaVNS after intervention (*p* < 0.05). Correction of *p*-values after post hoc analysis was made with FDR (Figure 4). However, there was no significant change after FDR (*p* > 0.05).

### 3.6. Adverse Events

There have been no reports of unexpected adverse events or serious adverse events associated with either in-ear or behind-ear TaVNS.

## 4. Discussion

It is stated that the vagus fibers in the ear are mostly found in the cymba concha part [21], but there are not enough data regarding the density and effectiveness of the vagus fibers behind the ear (compared to the inside of the ear). The effects of the fibers behind the ear on the brain and secondarily on the organs may be different. The purpose of this study is to compare using the specially designed electrodes designed to match the anatomical distribution of the vagus nerve in-ear and behind-ear the results related to HR, SBP, DBP, and HRV.

The main findings of this study showed a decrease in SBP and an increase in RMSSD, suggesting that in-ear TaVNS might be more effective than behind-ear TaVNS. No significant difference was observed in DBP, HR, or other HRV parameters (PNS index, SNS index, LF power, HF power) compared to baseline.

ANS plays an important role in the regulation of HR through vagal and sympathetic fibers, and the HR decreases with parasympathetic activation and increases with sympathetic activation [22,23]. The vagus nerve innervates the sinoatrial node and atrioventricular node and causes a decrease in HR [24]. TaVNS stimulates the nucleus tractus solitarii in the brainstem via vagal fibers, activating the parasympathetic effect and reducing systemic vascular resistance. This is thought to be the cause of the decrease in HR [25]. The vagus nerve helps to reduce HR by regulating the atrial refractory period by stimulating cholinergic muscarinic receptors at the cellular level in the atrium [26,27]. Antonino et al. (2017) investigated the effect of TaVNS on acute cardiac baroreflex activity sensitivity in a study of 13 healthy participants and found a significant decrease in HR in the TaVNS group compared to sham stimulation [28]. In the present study, a significant decrease in HR was observed in the in-ear TaVNS group, whereas no change was observed in the behind-ear TaVNS, in-ear sham, and behind-ear sham groups. The reason why in-ear TaVNS is more effective than behind-ear TaVNS in decreasing HR may be related to the greater density of vagus nerve afferent fibers in tragus and concha.

SBP and DBP are closely related to ANS activity and TaVNS causes a decrease in blood pressure by increasing the activation of parasympathetic activity [29]. It is possible to observe a sequential decrease in HR, SBP, and DBP after TaVNS [30]. Garcia et al. (2022) investigated the effects of vagus nerve stimulation (VNS) on twenty participants diagnosed with hypertension and found a decrease in HR, SBP, and DBP after VNS [31]. Rodrigues et al. (2021) showed that transcranial electrical current reduced sympathetic modulation and caused a significant decrease in SBP in individuals diagnosed with hypertension [32]. In the present study, a significant decrease in SBP and DBP after TaVNS was observed in both the in-ear and behind-ear TaVNS groups compared to baseline, whereas no change was observed in-ear sham and behind-ear sham stimulation groups. This shows that in-ear and behind-ear TaVNS methods are both effective in decreasing SBP and DBP by providing autonomic modulation.

RMSSD is defined as the root mean square of successive heartbeat differences, and a high RMSSD score indicates better health and lower stress levels than a low RMSSD score [33]. RMSSD is one of the HRV sub-parameters defined as an indicator of the parasympathetic response to stress and allows direct measurement of parasympathetic modulation via vagus nerve innervation [34]. Machetanz et al. (2021) stimulated the vagus nerve from different regions of the ear (cavum conchae, cymba conchae, outer tragus, inner tragus, crus helicis, and fossa triangularis) in healthy subjects and found that the cymba conchae and inner tragus caused an increase in HRV and RMSSD compared to other targets [35]. In the present study, an increase in RMSSD was observed after TaVNS in the in-ear and behind-ear groups compared to baseline, whereas no change was observed in the in-ear and behind-ear sham stimulation groups. When comparing in-ear and behind-ear TaVNS groups, a difference was observed, and it was found that in-ear TaVNS was more effective than behind-ear TaVNS. This may be related to the targeting of the inner tragus and cymba conchae in in-ear TaVNS. On the other hand, no significant difference was found between in-ear and behind-ear TaVNS groups in terms of RMSSD after FDR correction. This may be due to the small sample size.

LF and HF power, calculated from the mean length of beat-to-beat intervals (RR), express sympathetic and parasympathetic modulation respectively [36]. Vagal modulation is defined by the HF power and may be related to the outcome of increased parasympathetic activity. Although the LF power has previously been shown to represent predominantly sympathetic modulation, its representation of sympathetic modulation is controversial as it is associated with both sympathetic and parasympathetic fibers [37]. Szeles et al. (2021) investigated the effect of TaVNS on HRV in healthy subjects, TaVNS caused a significant increase in HRV, but no difference was found in LF and HF power [9]. In the study by Keute et al. (2021) investigating the change in HRV in healthy subjects according to the area where TaVNS was applied (cymba turbinate and tragus), no significant difference was found in LF/HF and HF parameters after TaVNS [1]. In the present study, no significant change was observed in LF and HF power after TaVNS compared to baseline. The reason for this may be that LF and HF power were not sufficient to measure the change in sympathetic and parasympathetic activity or that the flow parameters selected during TaVNS were insufficient to change LF and HF power.

This study has some limitations. The present study compared two different stimulation methods (area) and active-sham stimulation. As these stimulation methods and active-sham stimulation comparisons were performed with a relatively small number of participants, these comparisons need to be completed in the future with larger populations and more stimulation sessions. The validity of 5 min HRV measurements has been demonstrated, but the effects of TaVNS may be more strongly demonstrated with longer measurement times. Since this study was conducted in healthy adults aged 18-45 years, different results may be obtained in a population outside this age range, including those with chronic diseases associated with ANS dysfunction. The strength of the study is the comparison of in-ear and behind-the-ear stimulation methods with sham stimulation results.

## 5. Conclusions

In conclusion, TaVNS can reduce HR, SBP, and DBP and increase HRV when applied to specific auricular areas using various methods. In this context, in-ear TaVNS has been shown to be more effective than behind-ear TaVNS. Stimulating different regions with varying current parameters will help us to obtain more promising results in understanding the effects of TaVNS soon.

## Figures and Tables

**Table 1:** The physical characteristics of the groups.

	Group 1 In-Ear TaVNS	Group 2 Behind-Ear TaVNS	Group 3 In-Ear Sham	Group 4 Behind-Ear Sham		
Baseline	n = 19	n = 19	n = 19	n = 19		
Variables	Mean ± SD	Mean ± SD	Mean ± SD	Mean ± SD	F	*p*
Age (year)	26 ± 6.01	25 ± 5.86	26 ± 3.42	26 ± 4.78	0.242	0.867
Height (cm)	175 ± 0.59	171 ± 0.80	170 ± 1.13	174 ± 0.64	1.418	0.244
Weight (kg)	71 ± 6.94	73 ± 5.68	71 ± 7.45	68 ± 6.92	1.564	0.206
BMI (kg/m^2^)	31 ± 12.05	33 ± 7.99	43 ± 3.93	46 ± 9.04	1.250	0.345
Gender	n (%)	n (%)	n (%)	n (%)	X^2^	*p*
Male	10 (53%)	10 (53%)	10 (53%)	10 (53%)	2.521	0.343
Female	9 (47%)	9 (47%)	9 (47%)	9 (47%)		

Note: *p*-values are derived from the ANOVA, *p* > 0.05. Abbreviations: Standard deviation (SD), centimeters (cm), kilograms (kg), number of participants (n), chi-squared test (X^2^).

**Table 2:** Changes in HR, SBP, DBP, and HRV at baseline and after the TaVNS.

Variables	Baseline	after TaVNS		
HR (n = 19)	Mean ± SD	Mean ± SD	t	*p*
In-ear TaVNS	89.73 ± 14.18	82.26 ± 5.26	2.655	0.016 *
Behind-ear TaVNS	90.89 ± 13.15	87.78 ± 6.13	1.028	0.318
In-ear sham	92.68 ± 14.55	94.63 ± 9.46	0.706	0.140
Behind-ear sham	92.52 ± 10.25	91.84 ± 5.94	0.232	0.390
SBP (n = 19)	Mean ± SD	Mean ± SD	t	*p*
In-ear TaVNS	135.47 ± 11.90	118.57 ± 15.67	5.408	0.004 *
Behind-ear TaVNS	119.73 ± 15.12	107.01 ± 15.77	6.509	0.007 *
In-ear sham	124.47 ± 14.68	122.94 ± 13.02	0.627	0.200
Behind-ear sham	119.21 ± 15.36	118.02 ± 15.94	0.761	0.113
DBP (n = 19)	Mean ± SD	Mean ± SD	t	*P*
In-ear TaVNS	92.84 ± 13.95	81.78 ± 13.51	5.058	0.002 *
Behind-ear TaVNS	85.47 ± 8.44	77.26 ± 10.39	6.703	0.011 *
In-ear sham	87.57 ± 10.33	86.52 ± 11.46	0.131	0.352
Behind-ear sham	85.89 ± 8.53	87.47 ± 9.96	0.507	0.446
RMSSD (n = 19)	Mean ± SD	Mean ± SD	t	*p*
In-ear TaVNS	7.64 ± 1.78	9.76 ± 2.94	2.774	0.013 *
Behind-ear TaVNS	7.31 ± 2.23	8.59 ± 2.53	2.809	0.012 *
In-ear sham	9.76 ± 2.94	9.64 ± 1.78	0.774	0.130
Behind-ear sham	10.52 ± 3.44	9.89 ± 2.76	0.933	0.422
LF power (n = 19)	Mean ± SD	Mean ± SD	t	*p*
In-ear TaVNS	244.89 ± 95.51	122.47 ± 76.87	1.703	0.106
Behind-ear TaVNS	166.78 ± 80.42	104.52 ± 48.31	2.903	0.098
In-ear sham	223.65 ± 67.79	122.47 ± 76.87	1.703	0.117
Behind-ear sham	199.23 ± 75.77	202.98 ± 65.12	0.766	0.288
HF power (n = 19)	Mean ± SD	Mean ± SD	t	*p*
In-ear TaVNS	49.68 ± 82.88	31.84 ± 25.51	0.837	0.414
Behind-ear TaVNS	25.6 ± 13.57	30.78 ± 18.98	−0.854	0.404
In-ear sham	45.52 ± 66.32	30.52 ± 20.23	0.536	0.770
Behind-ear sham	34.08 ± 20.54	35.09 ± 22.09	0.551	0.118

Note: *p*-values are derived from paired samples *t*-test, *p* > 0.05, * *p* < 0.05. Abbreviations: Paired samples *t*-test (t), number of participants (n), standard deviation (SD), heart rate (HR), systolic blood pressure (SBP), diastolic blood pressure (DBP), transcutaneous auricular vagus nerve stimulation (TaVNS), low-frequency (LF), high-frequency (HF), root mean square of consecutive differences (RMSSD).

**Table 3:** Between groups comparison on HR, SBP, DBP, and HRV at baseline and after the TaVNS.

	In-Ear TaVNS Mean ± SD	Behind-Ear TaVNS Mean ± SD	In-Ear Sham Mean ± SD	Behind-Ear Sham Mean ± SD	F	*p*	Mean Square	Partial Eta Squared
HR								
Baseline	89.73 ± 14.18	90.89 ± 13.15	92.68 ± 14.55	91.46 ± 12.94	0.217	0.884		
After TaVNS	82.26 ± 5.26	87.79 ± 6.13	84.63 ± 9.46	86.84 ± 5.94	2.425	0.073	115.382	0.092
SBP								
Baseline	135.47 ± 11.99	119.74 ± 15.12	124.47 ± 14.68	119.21 ± 15.36	2.335	0.081		
After TaVNS	118.57 ± 15.67	107.00 ± 15.77	112.94 ± 13.02	108.01 ± 15.94	5.276	0.002 *	1082.294	0.180
DBP								
Baseline	92.84 ± 13.95	85.47 ± 8.44	87.57 ± 10.33	87.94 ± 10.75	1.957	0.128		
After TaVNS	81.78 ± 13.50	77.26 ± 10.39	80.52 ± 11.46	77.47 ± 9.96	0.738	0.533	96.140	0.030
RMSSD								
Baseline	7.64 ± 1.78	7.31 ± 2.23	9.76 ± 2.94	10.52 ± 3.44	0.182	0450		
After TaVNS	9.76 ± 2.94	8.59 ± 2.53	9.64 ± 1.78	9.89 ± 2.76	3.515	0.001 *	8.678	0.039
LF power								
Baseline	244.89 ± 95.51	166.78 ± 80.42	223.65 ± 67.79	199.23 ± 75.77	0.640	0.531		
After TaVNS	122.47 ± 76.87	104.52 ± 48.31	122.47 ± 76.87	202.98 ± 65.12	0.432	0.651	2040.018	0.016
HF power								
Baseline	49.68 ± 82.88	25.68 ± 13.57	45.52 ± 66.32	34.08 ± 20.54	0.786	0.461		
After TaVNS	31.84 ± 25.51	30.78 ± 18.98	30.52 ± 20.23	35.09 ± 22.09	0.013	0.987	7.018	0.028

Note: *p*-values are derived from ANOVA, *p* > 0.05, * *p* < 0.05. Abbreviations: Standard deviation (SD), heart rate (HR), systolic blood pressure (SBP), diastolic blood pressure (DBP), transcutaneous auricular vagus nerve stimulation (TaVNS), low-frequency (LF), high-frequency (HF), root mean square of consecutive differences (RMSSD).

**Table 4:** Post hoc analysis of RMSSD and SBP after TaVNS.

Variables	Rate of Change Difference	Std. Error	*p*	%95 CI	
				Lower Bound	Upper Bound
RMSSD					
In-ear TaVNS–Behind-ear TaVNS	0.33	0.631	0.046 *	−1.19	1.85
In-ear TaVNS–In-ear sham	−2.00	0.631	0.990	−1.52	1.30
In-ear TaVNS–Behind-ear sham	−2.25	0.631	0.764	−2.07	1.83
Behind-ear TaVNS–In-ear sham	−2.33	0.631	0.260	−2.57	1.33
Behind-ear TaVNS–Behind-ear sham	−2.58	0.631	0.726	−2.45	1.45
In-ear sham–Behind-ear sham	−0.25	0.631	0.810	−1.45	2.45
SBP					
In-ear TaVNS–Behind-ear TaVNS	11.58	4.91	0.044 *	−1.34	24.50
In-ear TaVNS–In-ear sham	5.63	4.91	0.663	−7.29	18.55
In-ear TaVNS–Behind-ear sham	10.57	4.91	0.147	−2.34	23.50
Behind-ear TaVNS–In-ear sham	−5.94	4.91	0.623	−18.87	6.98
Behind-ear TaVNS–Behind-ear sham	−1.00	4.91	0.997	−13.92	11.92
In-ear sham–Behind-ear sham	4.94	4.91	0.746	−7.98	17.87

Note: *p*-values are derived from ANOVA, *p* > 0.05, * *p* < 0.05. Abbreviations: Standard deviation (SD), transcutaneous auricular vagus nerve stimulation (TaVNS), systolic blood pressure (SBP), confidence interval (CI), standard (Std).

## Data Availability

Data are available on request from the authors.
